# Prevalence of Fetal Alcohol Syndrome and Maternal Characteristics in a Sample of Schoolchildren from a Rural Province of Croatia

**DOI:** 10.3390/ijerph10041547

**Published:** 2013-04-16

**Authors:** Giorgie Petković, Ingeborg Barišić

**Affiliations:** 1 Children’s Hospital Srebrnjak, Srebrnjak 100, Zagreb 10000, Croatia; 2 Children’s Hospital Zagreb, Medical School University of Zagreb, Klaićeva 16, Zagreb 10000, Croatia; E-Mail: ingeborg.barisic@kdb.hr

**Keywords:** FAS, prevalence, schoolchildren, rural, Croatia

## Abstract

Fetal alcohol syndrome (FAS) is a congenital syndrome caused by maternal alcohol consumption during pregnancy and is entirely preventable by abstinence from alcohol drinking during this time. Little is known about the prevalence of FAS and maternal alcohol consumption during pregnancy in Western countries. We present the results of FAS/partial fetal alcohol syndrome (PFAS) prevalence study and maternal characteristics in a sample of schoolchildren from a rural province of Croatia. This study involved seven elementary schools with 1,110 enrolled children attending 1st to 4th grade and their mothers. We used an active case ascertainment method with passive parental consent and Clarified IOM criteria. The investigation protocol involved maternal data collection and clinical examination of children. Out of 1,110 mothers, 917 (82.6%) answered the questionnaire. Alcohol exposure during pregnancy was admitted by 11.5%, regular drinking by 4.0% and binge drinking by 1.4% of questioned mothers. Clinical examination involved 824 (74.2%) schoolchildren and disclosed 14 (1.7%) with clinical signs of FAS and 41 (5.0%) of PFAS. The observed FAS prevalence, based on 74.2% participation rate, was 16.9, PFAS 49.7 and combined prevalence was 66.7/1,000 examined schoolchildren. This is the first FAS prevalence study based on active ascertainment among schoolchildren and pregnancy alcohol drinking analysis performed in a rural community of Croatia and Europe. High prevalence of FAS/PFAS and pregnancy alcohol consumption observed in this study revealed that FAS is serious health problem in rural regions as well as a need to develop future studies and preventive measures for pregnancy alcohol drinking and FASD.

## 1. Introduction

Fetal alcohol syndrome (FAS) is a congenital syndrome caused by maternal alcohol intake during pregnancy. Clinical manifestations of the syndrome include combination of characteristic dysmorphic facial features, low height and weight, small head circumference, structural and functional anomalies of the central nervous system and possibly other organ disorders [1,2,3,4]. This syndrome is the most common preventable cause of mental retardation and lifelong disabilities such as learning difficulties, poor academic achievements, behavioral problems, inadequate social relationships and inability of independent living [5,6]. The clinical manifestations are well documented, however epidemiology of FAS and partial fetal alcohol syndrome (PFAS) are still a matter of intensive studies because precise data on maternal risk factors and FAS prevalence are essential for the development of appropriate preventive programs.

Investigations of FAS epidemiology and contributing risk factors are limited and the results of these studies are variable [4]. FAS prevalence variability is attributed to racial differences, ethnic groups, social-economic and cultural factors [7]. Literature data revealed that methods used in epidemiology studies are significant source of variability. Different investigation approaches yield different frequency rates. Active case ascertainment among schoolchildren is suitable for epidemiologic studies of FAS. Schoolchildren are the representatives of the entire local population and are of age when the dysmorphic facial features are apparent, making FAS diagnosis more accurate [8]. Due to reduced selectivity, active ascertainment produces higher FAS prevalence rate than record review or clinical base studies [8,9]. Most studies based on active ascertainment among schoolchildren were performed in the USA and high risk community of South Africa [8]. However, studies in Europe are rare and involved the Italian Lazio region and an urban sample of schoolchildren in Croatia [10,11,12]. Observed FAS prevalence is variable and ranges from 3.1 to 74.2 per 1,000 schoolchildren [8]. In this paper we present the results of active case ascertainment study of FAS/PFAS prevalence and maternal characteristics among primary schoolchildren. The aim of this study was to evaluate maternal risk factors and FAS/PFAS prevalence in a Croatian rural province. 

## 2. Experimental Section

In this study we used the active case ascertainment method to evaluate FAS and PFAS prevalence among schoolchildren attending 1st to 4th grade elementary school and the pregnancy alcohol drinking habits of their mothers. The study did not include children attending specialized institutions and modified education curriculum. The study involved a province in the rural Northern region of Croatia. Most of the population lived in villages and the province’s capital, with 10,000 inhabitants, was the sole town. The province is famous for grape growing and wine making and the region had the highest alcohol consumption among women in Croatia [13]. In this study we focused on the province’s capital and all six of the surrounding counties. There were eight elementary schools in the index area, but one school refused cooperation, therefore, seven schools were involved in the study. One school was located in the province’s capital and six others in surrounding counties. All seven of the investigated schools had subordinated district schools located in smaller villages. In all, seven elementary and 13 district schools were included in this study. The total number of children attending 1st to 4th grade in these schools was 1,110. 

The investigation protocol involved maternal data collection based on a self-completed questionnaire and clinical examination of their children. In accordance with school directors, parents were informed of the upcoming research by sending notices to their homes. The notices included data on the study goals, methods, guarantee of confidentiality and a questionnaire for the mothers to complete. The questionnaire had a total of 32 questions, it was written at an elementary grade level, and included questions related to demographic variables, health, nutrition, smoking and drinking habits. Items covering alcohol drinking habits were integrated among questions related to life style and dietary data. We investigated alcohol drinking habits for the last three months to define current alcohol consumption, and also alcohol drinking habits for the index pregnancy period. Amount of alcohol drinks per week as well as number of binge episodes were questioned. Trimester and type of alcohol used during pregnancy was also asked for. A short definition of an alcohol drink and binge was given in order to standardize the answers. Alcohol drink was defined as 0.5 dL of spirits, 1.5 dL of wine or 3.3 dL of beer, as derived from ounce metric system, and binge drinking as having five or more alcoholic drinks per occasion [14,15]. 

We used passive parental consent for the clinical part of the study [16]. The physician involved in the clinical part of the study was blinded to all information regarding data from maternal questionnaire. The evaluation of body mass and height, head circumference, palpebral fissure length, philtrum smoothness and upper lip thinness was performed by an experienced pediatrician [17,18,19,20]. Palpebral fissure length was measured in millimeters from endocathion to exocanthion in Frankfort plain by clear ruler [17]. Upper lip thinness and philtrum smoothness were evaluated using a five point lip-philtrum guide [17]. The anthropometric measures were evaluated using age specific percentile [18,19,20]. Diagnosis of FAS and PFAS was made using Clarified IOM criteria and we evaluated the combination of characteristic growth and facial features [21]. For the diagnosis of FAS, with or without confirmed maternal alcohol exposure, a child must exhibit a combination of growth retardation (height or weight at or under the 10th centile for age), microcephaly (head circumference at or under the 10th centile for age) and at least two characteristic facial anomalies (palpebral fissure length at or below the 10th centile for age, smooth philtrum as diagnosed by class 4 or 5, thin upper lip class 4 or 5 on lip-philtrum guide). The diagnosis of PFAS, with or without confirmed maternal alcohol exposure, required evidence of at least two cardinal facial features and one of following features: growth retardation or microcephaly [21]. The study protocol was approved by the Ethic committee of the Children’s Hospital Zagreb, Zagreb Medical School and Croatian Ministry of Science, Education and Sports. 

## 3. Results

### 3.1. Maternal Characteristics

This study involved seven elementary schools with 1,110 enrolled children and their mothers. The involved mothers showed high interest in the study and a high participation rate was obtained. Out of 1,110 children enrolled in the studied schools, the mothers of 917 (82.6%) answered the questionnaire. Maternal age spanned from 24 to 53 years and the mean age was 35.3 ± 4.9 years. The summary of the results is presented in Table 1. Elementary and high school education was reported by 722 (78.8%) mothers, 253 (27.6%) were unemployed and 39 (4.3%) mothers lived without a partner. Current smoking was reported by 188 (20.5%), while 91 (9.9%) mothers reported smoking during pregnancy. Special attention was paid to alcohol drinking habits. Current alcohol consumption was reported by 167 (18.2%), regular alcohol drinking by 145 (15.8%) and binge drinking by 53 (5.7%) out of the 917 mothers. No alcohol drinking in the last three months was reported by 728 (79.4%) mothers and 22 (2.4%) did not answer the related questions. Alcohol exposure during pregnancy was admitted by 105 (11.5%), regular drinking by 37 (4.0%) and binge drinking by 13 (1.4%) out of 917 mothers. Beer was drank by 69 and wine by 38 mothers and only one mother reported drinking spirits during pregnancy. Data analysis revealed that 17 (1.9%) mothers did not answer the question related to pregnancy alcohol consumption. Seven mothers answered that alcohol has beneficiary effects on pregnancy and 26 mothers did not answer the related question. 

**Table 1:** Characteristics of the 917 interviewed mothers.

Variable	Number	%
**Education level**		
Elementary school	30	3.3
High school	692	75.5
College	63	6.9
University	47	5.1
Unanswered	85	9.3
**Employment status**		
Unemployed	253	27.6
Employed	660	72.0
Unanswered	4	0.4
**Marital status**		
Living with a partner	872	95.1
Not living with partner	39	4.3
Unanswered	6	0.7
**Tobacco use**		
Current smokers	188	20.5
Pregnancy smokers	91	9.9
Current alcohol consumption	145	15.8
1–3 drinks per week	139	15.2
3–7 drinks per week	6	0.7
Current binge drinking	53	5.7
1× in the last 3 months	23	2.5
2–3× in the last 3 months	24	2.6
>4× in the last 3 months	6	0.7
Current alcohol consumption admitted	167	18.2
Weekly alcohol consumption	114	12.4
Weekly alcohol consumption and binge drinking	31	3.4
Binge drinking	22	2.4
Current alcohol consumption negative	728	79.4
Current alcohol consumption unanswered	22	2.4
**Attitude towards alcohol effect on pregnancy outcome**		
Positive	7	0.8
Not answered	26	2.8
Negative	884	96.4
Pregnancy alcohol consumption	37	4.0
1–3 drinks per week	37	4.0
3–7 drinks per week	0	0
Pregnancy binge drinking	13	1.4
1× during pregnancy	2	0.2
2–3× during pregnancy	11	1.2
>4× during pregnancy	0	0
**Type of alcohol consumed during pregnancy**		
Beer	64	6.9
Wine	33	3.6
Spirits	1	0.1
Beer + wine	5	0.5
Beer + spirits/wine + spirits/beer + wine + spirits	0	0
**Trimester of alcohol consumption**		
1st trimester	26	2.8
2nd trimester	7	0.8
3rd trimester	12	1.3
Entire pregnancy	25	2.7
Pregnancy alcohol consumption admitted (positive answer to one or more pregnancy alcohol consumption questions)	105	11.5
Pregnancy alcohol consumption negative (negative answer to all pregnancy alcohol consumption questions)	795	86.7
Pregnancy alcohol consumption unanswered (all pregnancy alcohol consumption questions unanswered)	17	1.9

### 3.2. Characteristics of Examined Schoolchildren

Out of 1,110 children, 826 (74.4%) received the parental consent, but two children were unavailable for clinical examination, so a total of 824 (74.2%) children were examined for the clinical symptoms of FAS. The sample included 423 (51.3%) boys and 401 (48.7%) girls with ages spanning from 7.0 to 11.9 years and average age of 9.0 ± 1.2 years. The performed investigation revealed growth retardation in 161 (19.5%) and head circumference at or below the 10th centile for age in 79 (9.6%) children. Two FAS facial features were present in 172 (20.9%) and three facial features in 77 (9.3%) examined children (Table 2). 

**Table 2:** Clinical characteristics in a sample of 824 examined children.

Variable	Number	%
Male	423	51.3
Female	401	48.7
Growth retardation (height and/or weight ≤10th centile)	161	19.5
Head circumference ≤10th centile	79	9.6
2 FAS facial features	172	20.9
3 FAS facial features	77	9.3
FAS	14	1.7
FAS with confirmed alcohol exposure	4	0.5
FAS with negative alcohol exposure	10	1.2
FAS with unanswered alcohol exposure	0	0
PFAS	41	5.0
PFAS with confirmed alcohol exposure	6	0.7
PFAS with negative alcohol exposure	31	3.7
PFAS with unanswered alcohol exposure	4	0.5

### 3.3. FAS/PFAS Group of Children

Out of 824 examined children, 55 (6.7%) children had clinical signs of FAS or PFAS. The group consisted of 32 boys and 23 girls, with ages spanning from 7.1 to 11.5 years. In all, 14 (1.7%) pupils had clinical signs of FAS and 41 (5.0%) schoolchildren had clinical signs of PFAS. A total of 10 diagnosed children had confirmed alcohol exposure during pregnancy, including four children with FAS and six children with PFAS (Table 2). The remaining FAS/PFAS children did not have confirmed alcohol exposure during pregnancy as stated by mothers in the questionnaire. 

In this study we compared maternal characteristics of 55 children with features of FAS and PFAS and a group of 769 children without sign of FAS/PFAS. Data are presented in Table 3. No significant differences between the two groups were observed for age, education level, employment, marital status, current alcohol intake and smoking during pregnancy. The frequency of confirmed pregnancy alcohol consumption was significantly higher in FAS/PFAS group (18.2%) when compared to non-FAS/PFAS group (10.4%). The analysis revealed significantly higher frequency of alcohol drinking during the entire pregnancy and the last trimester in a group of FAS/PFAS mothers compared to non-FAS/PFAS group. 

**Table 3:** Demographic and substance use by mothers of examined children with and without features of FAS/PFAS.

Maternal characteristics	Children without FAS/PFAS		Children with FAS/PFAS		*p*
	N = 769		N = 55		
	N	%	N	%	
Maternal age					
<35 years	421	54.7	32	58.2	0.855 (NS)
35–45 years	305	39.7	20	36.4	
>45 years	18	2.3	1	1.8	
unanswered	25	3.3	2	3.6	
Education level					
elementary school	27	3.5	0	0.0	0.089 (NS)
high school	580	75.4	46	83.6	
college	56	7.3	0	0.0	
university	39	5.1	3	5.5	
unanswered	67	8.7	6	10.9	
Employment status					
employed	558	72.6	36	65.5	0.243 (NS)
unemployed	209	27.2	19	34.5	
unanswered	2	0.3	0	0.0	
Marital status					
living with partner	731	95.1	53	96.4	0.778 (NS)
living without partner	34	4.4	2	3.6	
unanswered	4	0.5	0	0.0	
Current alcohol consumption					
admitted	141	18.3	9	16.4	>0.05 (NS)
negative	614	79.8	42	76.3	
unanswered	14	1.8	4	7.2	
Smoking during pregnancy					
admitted	75	9.8	5	9.1	0.943 (NS)
negative	681	88.6	47	85.5	
unanswered	13	1.7	3	5.5	
Pregnancy alcohol consumption					
admitted	80	10.4	10	18.2	0.001
negative	679	88.3	41	74.5	
unanswered	10	1.3	4	7.3	
Alcohol consummation period					
1st trimester	21	2.7	0	0.0	<0.001
2nd trimester	6	0.8	0	0.0	
3rd trimester	5	0.7	4	7.3	
entire pregnancy	19	2.5	3	5.5	

(NS = not significant).

### 3.4. FAS/PFAS Prevalence Rate

Based on 74.2% participation rate, observed prevalence was 16.9/1,000 for FAS and 49.7/1,000 schoolchildren for PFAS. Total observed combined rate of FAS/PFAS in the studied sample was 66.7/1,000 examined schoolchildren. FAS/PFAS prevalence with confirmed pregnancy alcohol exposure was 12.1 per 1,000 schoolchildren. 

## 4. Discussion

### 4.1. Participation Rate

In this study we obtained a high participation rate of 82.6% for maternal data evaluation and 74.2% for the clinical examination. These rates were higher compared to children inclusion rate of 50% in the Italian sample and to the participation rate of 63.0% and inclusion rate of 51.0% in our previous study on the urban sample of schoolchildren [10,12]. In the urban sample the higher inclusion rate (53%) was obtained when a physician explained the project to the parents, while the lower inclusion rate (47%) was obtained when written notices were sent to mothers. Thus the difference in the participation and inclusion rates between the two studies were not due to the different parental information process but can be attributed to the different characteristics of the investigated populations. However, we speculate that personal contact between the medical researcher and parents would have resulted in even better rates in the present study.

### 4.2. Risk Factors for Pregnancy Alcohol Consumption

Earlier studies disclosed a variety of risk factors for maternal pregnancy alcohol consumption in rural communities [22]. In this study we evaluated maternal habits and characteristics such as current alcohol consumption, age, smoking, education level, employment and marital status. Our group of questioned mothers had a lower mean age when compared to other published studies in Europe [10,11,12]. Living without a partner was rare (4.3%), as expected in the highly traditional rural setting, while higher frequencies (17.5%) were reported in Italian study [8]. Almost a third (27.6%) of the mothers was unemployed, in contrast to 40.3% in the Italian study [10]. Educational achievement was low in our sample of mothers, and only 12.0% of the questioned women had a college or university diploma when compared to 55.3% of women in the capital city of Zagreb and 19.7% in the Italian Lazio sample [11,12]. In this study current smoking frequency of 20.5% was lower to that reported in the Italian (57.7%) and Croatian urban samples (24%) [11,23], while it was higher in comparison to the 10.54% reported in the general female population of the rural Northern region [24]. In the present study alcohol consumption during the last three months was admitted by 18.2%, binge drinking by 5.7% of questioned mothers and none admitted drinking more than seven drinks a week. These drinking rates were higher when compared to a Croatian study performed in 2009, which revealed that the frequency of heavy alcohol consumption (≥7 drinks a week or ≥6 drinks in a binge) among women of general population in the Northern rural region was 1.45% [13]. The differences in current drinking and smoking rates between studies suggest that drinking and smoking was more frequent in younger childbearing women when compared to the women of the general population in the same area. 

### 4.3. Alcohol Consumption during Pregnancy

In our sample of mothers admitted pregnancy drinking was lower when compared to other studies reported so far. Epidemiologic studies performed in USA, France, Ireland, Italy and Denmark showed that pregnancy alcohol consumption ranged from 12.4% to 53.9%, while binge drinking ranged from 3.4% to 26% [25,26,27,28,29,30]. Admitted pregnancy alcohol consumption in the present study was lower when compared to our previous study on the urban sample where 15.4% of mothers admitted drinking alcohol during pregnancy and 3.1% had at least one binge drinking episode [12]. Our findings were similar to published data which showed that 12.2% of US women confessed alcohol consumption during pregnancy and 1.9% of the interviewed pregnant women were binge drinkers [31]. The present study revealed marked differences between admitted pregnancy alcohol consumption and attitudes towards alcohol effects on pregnancy. In Croatia 97% of urban mothers believed that alcohol consumption has harmful effects during pregnancy and the frequency is almost the same in present study [12]. Several mothers mentioned the beneficial effects of alcohol on pregnancy outcome and they believed that drinking red wine during pregnancy would prevent anemia or drinking beer towards the end of pregnancy would increase lactation. It was interesting to note a 2-fold decrease in current smoking from 20.5% to 9.9% during pregnancy while alcohol consumption decreased to a much lesser extent, with a drop from 18.2% to 11.5%. These observations were in accordance with the results of an Italian study performed in 2007, where 68.4% of women reduced or stopped smoking, but only 21.5% reduced or stopped drinking during pregnancy [27]. These data suggested that women were not appropriately informed and were not aware of the toxic effects of alcohol on pregnancy outcome and considered smoking more noxious to fetus development compared to alcohol exposure. 

### 4.4. Comparison of Maternal Characteristics between FAS/PFAS and Non-FAS/PFAS Group

Of interest were data on maternal characteristics in FAS/PFAS and non-FAS/PFAS groups of children. Significant differences between the groups were detected for pregnancy alcohol consumption. Confirmed pregnancy alcohol consumption in the FAS/PFAS group was higher (18.2%) to observed frequency in the whole sample of questioned mothers (11.5%) and significantly higher when compared to non-FAS/PFAS mothers (10.4%). Contrary to our observation, reported rates of overall drinking during pregnancy between groups of mothers were not significantly different in the Italian sample [10]. However, like in the Italian sample, a significantly higher frequency of alcohol consumption in the 3rd trimester was admitted by FAS/PFAS mothers compared to non-FAS/PFAS group [11]. Our data suggest that the mothers of non-FAS/PFAS children changed their drinking habits during pregnancy, and stopped drinking once they found out they were pregnant, while mothers of FAS/PFAS children did not. In fact, in the non-FAS/PFAS group there was reduced frequency of alcohol consumption in the 2nd and the 3rd compared to 1st trimester, pregnancy alcohol consumption was 7.9% lower than reported current consumption and there was higher rate of denial of pregnancy drinking compared to current consumption. On the other hand, in the FAS/PFAS group alcohol consumption during entire pregnancy, in all trimesters, was more than twice as frequent as in non-FAS/PFAS group. In addition there was no drop in frequency of pregnancy alcohol consumption compared to current drinking, and no differences in the rate of denial of current and pregnancy drinking. These data suggested that the women who had a child in the FAS or PFAS group did not change drinking habits once they found out they were pregnant, and consumed alcohol during the entire pregnancy. The comparison of maternal data in FAS/PFAS and non-FAS/PFAS group revealed no significant differences in smoking habits and our results thus confirmed previous investigation in an Italian Lazio sample of schoolchildren [11]. Analysis of FAS/PFAS group of children revealed higher frequency of low maternal education (83.6%) compared to non-FAS/PFAS mothers (78.9%), as well as compared to the education level of FAS/PFAS mothers in the Lazio study, where 77% of mothers had elementary and high school education [11]. 

### 4.5. FAS/PFAS Prevalence Studies

Nine studies of FAS/PFAS/FASD prevalence based on active case ascertainment among schoolchildren have been completed and most of these studies used clinical criteria proposed by the revised IOM classification [21]. Several studies have been performed in a rural high risk population of South Africa and reported a FAS prevalence range from 46.4 to 74.2/1,000 schoolchildren, while FAS/PFAS prevalence was reported as high as 89.2/1,000 schoolchildren [32,33,34,35]. These findings constitute the high end of FAS frequency reported among schoolchildren. Lower FAS prevalence were observed in USA and reported as 3.1 and 4.4/1,000 children [9,16]. FASD prevalence in two wave urban and suburban studies in USA ranged from 9.5 to 24.8 per 1,000 schoolchildren [8]. In Europe FAS prevalence investigations are rare, and involved two wave studies in the Italian Lazio region and a Croatian urban schoolchildren sample [10,11,12]. The Italian Lazio study, published in 2011, reported FAS prevalence of 4.0–12.0/1,000, PFAS 18.1–46.3/1,000 and FASD 23.1–62.6/1,000 schoolchildren [11]. The second European active ascertainment study was performed in Croatia on an urban sample of schoolchildren and the reported rate of FAS was 6.44/1,000, of PFAS 34.33/1,000 and the combined rate was 40.77/1,000 schoolchildren [12]. 

**Figure 1:**
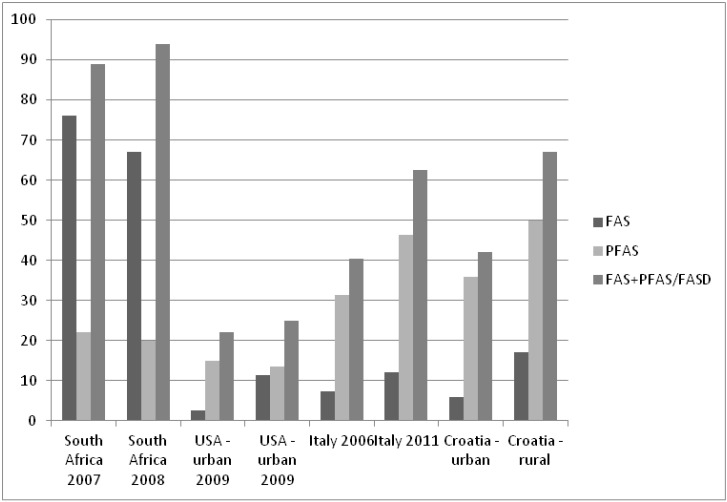
FAS, PFAS and combined prevalence in our studies and so far performed investigations reported in literature.

The present investigation revealed a high FAS/PFAS prevalence and we speculate that the observed prevalence reflected a high risk rural population from a wine growing region. The prevalence was higher when compared to the Croatian urban sample study and similar to the high end estimates of the Lazio study, where only 20.6% of women had a rural residency [10]. This investigation also confirmed previous observations of variable relationships between FAS, PFAS and FASD among different investigated populations [11,36]. Investigations performed in South Africa revealed a higher prevalence of FAS, while studies in Europe, including the present analysis, revealed higher PFAS frequency (Figure 1). These differences could be attributed to different habits, pattern and quantity of alcohol consumption. Heavy drinking patterns produced predominantly severe fetus damage and FAS, while the toxic effects of lower consumption rates observed in Italy and Croatia were associated with PFAS and another entity of the spectrum. Literature data suggests that the clinical consequences of pregnancy alcohol consumption were more complex and caused by poorly understood interactions of the amount of consumed alcohol and different protective factors and factors that promote the toxic effects of alcohol on the fetus, such as genetic factors, nutrition and maternal alcohol metabolism [37,38,39].

### 4.6. Study Limitations

Limitations of this study included maternal recall bias regarding pregnancy alcohol intake and the use of a self-reported questionnaire with the potential of underestimating alcohol consumption during pregnancy. Furthermore, the study did not include children attending specialized institutions and modified education curriculum, with the possibility of underestimating FAS/PFAS prevalence. A third limitation included evaluated features for PFAS. This study focused on FAS and PFAS, the two most severe entities of FASD, and the Revised IOM diagnostic criteria were used [21]. In the Revised IOM, criteria for PFAS include two or more facial anomalies and one of the following characteristics: growth retardation, microcephaly, and complex pattern of behavioral/cognitive abnormalities [21]. Due to the goal of the study, time limitations, and to ensure minimal disruption of the teaching process, we did not evaluate the children for behavioral problems and cognitive abnormalities. Therefore we may have missed some children with PFAS and other entities of FASD. This limitation may explain some of the differences between our results and other data reported so far in the literature. A fourth limitation was the lack of measurement of biomarkers of prenatal exposure to maternal alcohol intake, an approach that provides more accurate information on maternal drinking habits during pregnancy [40].

### 4.7. Future Studies

Data on FASD prevalence, maternal risk factors and pregnancy drinking habits in Europe are limited and information for the development of efficient national FASD preventive programs is still inadequate. Investigations performed so far focused on FAS and PFAS, the two most severe forms of FASD, and data on prevalence of alcohol-related birth defects (ARBD) and alcohol-related neurodevelopment disorder (ARND) are few [11]. Additional studies that integrate the evaluation of complex pattern of behavioral problems, cognitive abnormalities and congenital structural defects are needed for accurate estimation of FASD prevalence and the magnitude of this preventable health problem in Croatia and Europe. A possible future step in FASD prevalence studies and diagnosis is the use of biomarkers of alcohol consumption during pregnancy [41]. Recent investigations used this objective assessment of prenatal alcohol consumption and revealed that gestational ethanol exposure is widespread in Italy and Spain.

## 5. Conclusions

In summary, results of this study, based on a 74.2% participation rate, revealed a FAS prevalence of 16.9, a PFAS one of 49.7 and a combined prevalence of 66.7 per 1,000 schoolchildren. FAS/PFAS prevalence with confirmed alcohol exposure was 12.1 per 1,000 schoolchildren. Pregnancy alcohol drinking was admitted by 11.5% of mothers and 1.4% confirmed pregnancy binge drinking. FAS epidemiology studies in Europe are few, and this is the first study performed in a high risk rural region. Detected high FAS prevalence rates and observed differences in pregnancy alcohol consumption among investigations performed so far in Europe emphasize the need for further epidemiologic studies. These studies will contribute to our understanding of the relationship between drinking patterns and clinical consequences, as well as development of diagnostic procedures and programs that will increase awareness of harmful alcohol effects on pregnancy outcome among women in Europe. 
